# Cardiovascular Health and Diet Quality among Vegetarians, Vegans and Omnivores: Insights from a Large Urban Population in Poland

**DOI:** 10.3390/nu16203438

**Published:** 2024-10-10

**Authors:** Oliwia Grygorczuk, Martyna Mrozik, Anna Lipert, Sylwia Kamińska, Adam Białas, Wojciech Drygas, Ewa Rębowska, Stanisław Łęgocki, Anna Jegier, Katarzyna Szmigielska, Magdalena Kwaśniewska

**Affiliations:** 1 Lifestyle Medicine Students’ Scientific Association at the Department of Preventive Medicine, Medical University of Lodz, 90-752 Lodz, Poland; oliwia.grygorczuk@student.umed.lodz.pl (O.G.); sylwia.smarzych@student.umed.lodz.pl (S.K.); 2 Department of Preventive Medicine, Medical University of Lodz, 90-752 Lodz, Poland; martyna.mrozik@umed.lodz.pl (M.M.); anna.lipert@umed.lodz.pl (A.L.); wojciech.drygas@umed.lodz.pl (W.D.); ewa.rebowska@umed.lodz.pl (E.R.); stanislaw.legocki@umed.lodz.pl (S.Ł.); 3 Department of Pneumology, Medical University of Lodz, 90-419 Lodz, Poland; adam.bialas@umed.lodz.pl; 4 Department of Sports Medicine, Medical University of Lodz, 92-213 Lodz, Poland; anna.jegier@umed.lodz.pl (A.J.); katarzyna.szmigielska@umed.lodz.pl (K.S.)

**Keywords:** dietary habits, plant-based diet, vegetarian diet, vegan diet, cardiovascular risk, Poland

## Abstract

**Background/Objectives:** Dietary habits are among the most significant determinants of health. The aim of this study was to assess the nutritional quality and cardiovascular profiles of individuals following plant-based diet. **Methods:** The study population comprised 199 individuals (136 women, 63 men; mean age 33.9  ±  8.9 years) including vegans (VG; *n* = 50), vegetarians (VN; *n* = 101) and omnivores (OV; *n* = 48). In this analysis the following procedures were assessed: a questionnaire interview, anthropometric and blood pressure measurements, and a blood sample collection. Dietary patterns were evaluated using the Food Frequency Questionnaire and a 24-h dietary recall. **Results:** Vegans exhibited the lowest protein intake relative to the other groups (*p* < 0.05) and a significantly higher intake of polyunsaturated fatty acids and lower intake of cholesterol compared to VN and OV (*p* < 0.05). Vegans had significantly lower levels of serum cholesterol, LDL cholesterol, and triglycerides, fasting glucose and high-sensitivity C-reactive protein (*p* < 0.05). No cases of overweight or obesity were observed among VN and VG participants. No instances of impaired fasting glucose or elevated blood pressure were noted among vegans. Hypercholesterolemia was identified in 56.2% of OV, 26.7% in VN and 16.0% in VG (*p* < 0.05), elevated blood pressure was recorded in one vegetarian and in 6.2% of OV participants. **Conclusions:** Our research indicates that plant-based diets are associated with a better cardiovascular profile compared to traditional diets. Moreover, suboptimal intake of essential nutrients, underscores the need for more effective public health interventions and improved nutrition education regardless of dietary patterns.

## 1. Introduction

Dietary habits are among the most significant determinants of health. A robust body of evidence underscores the critical role of optimal nutrition in reducing the incidence and mortality associated with chronic diseases, particularly cardiovascular disease (CVD), metabolic disorders, and cancers [1,2,3,4]. The World Health Organization, the EAT-Lancet Commission, and other leading health organizations globally advocate for well-balanced diets rich in minimally processed plant-based foods and reduced intake of animal products as a means to achieve substantial health benefits [5,6]. The preponderance of scientific literature focuses on the effects of plant-based dietary patterns on cardiovascular health, including their influence on risk factors, myocardial perfusion, and the progression of atherosclerosis [7,8,9,10,11,12]. The mechanisms by which plant-based diets confer cardiovascular protection are multifaceted and not yet fully characterized. Importantly, the positive health outcomes associated with such dietary patterns are not solely attributed to the reduction in animal product consumption but also numerous highly effective bioactive components like fiber, polyphenols or certain vitamins. Nevertheless, the majority of studies, including randomized controlled trials, have demonstrated that the cardiovascular benefits are predominantly derived from the overall dietary pattern rather than from isolated nutrients [13].

Epidemiological data indicate substantial changes in dietary patterns over the past few decades, with a clear trend towards plant-based diets [14]. For example, between 2014 and 2018, the number of Americans adhering to a vegan diet increased by 600% [15]. In the United Kingdom, the prevalence of vegetarianism among teenagers was reported to be between 6% and 11% [16]. In developed countries, the adoption of vegetarian diets is more common among women, individuals with higher education or socioeconomic status, those under 40 years old, and those with lower likelihood of having children. Conversely, in developing countries, vegetarianism is often associated with lower socioeconomic status [17].

Despite Poland’s long tradition of a high-fat diet rich in animal-derived products, plant-based diets have gained popularity in recent years. According to CEOWORLD magazine’s Consumer Insights for 2024, Poland ranks 19th globally in terms of its vegetarian and vegan population, at 8.4% and 1.8%, respectively [18]. This represents a significant increase from 2014, when only 1% of Poles were vegetarians according to CBOS data [19]. However, data on the prevalence of plant-based diets in Poland remain limited, highlighting the need for further investigation. The Central Statistical Office’s “Agriculture 2019” report notes a 2% decrease in average per capita meat consumption in Poland [20].

National surveys conducted post-pandemic by private enterprises suggest that approximately 20% of Polish consumers reassessed their dietary habits during the COVID-19 lockdown and found plant-based diets more appealing, leading to increased sales of meat alternatives [21]. A study by the IMAS Institute involving 1018 individuals aged 18–74 revealed that a shift towards plant-based diets is particularly common among young adults aged 18–24, with over half of this age group not consuming meat. Vegetarians and vegans in Poland are more likely to be female, highly educated, employed full-time with higher incomes, and residing in the southwestern regions of the country. Despite these trends, 61% of the study population reported neither purchasing nor consuming plant-based products [22]. The Kukuła Healthy Food 2020 report, based on a survey of catering customers, found that the highest prevalence of vegans was among individuals aged 25–34, while the highest prevalence of vegetarians was among those aged 18–24. Additionally, there is a consensus among various reports that flexitarianism—a dietary approach characterized by occasional consumption of meat within a predominantly plant-based diet—is on the rise [23]. According to a report of the Heinrich Böll Foundation in Warsaw 44% of Poles aged 15–29 reported reducing their meat consumption and incorporating more plant-based meals into their daily diet [24].

The term “plant-based diet” generally refers to dietary patterns with low frequency of animal food consumption. Depending on the specific type, it can encompass a variety of dietary approaches [25]. A typical vegetarian diet includes plant-based foods while excluding all forms of animal meat, including fish, mollusks, and crustaceans, though it may permit the consumption of dairy products, eggs and honey. The extreme variation of vegetarian diet is veganism, which excludes all animal products [17]. According to the Academy of Nutrition and Dietetics, well-planned vegetarian diets are safe for people of all ages and physiological conditions [26]. However, poorly balanced diets can lead to nutrient deficiencies and increase the risk of malnutrition, underscoring the need for studies not only on clinical outcomes but also on the nutritional quality of these dietary models.

Most comprehensive research in this area has been conducted in Western countries, with European representation from Finland, Switzerland, Germany and the United Kingdom [27,28,29,30,31,32]. There is a scarcity of studies examining adherence to current dietary guidelines and cardiovascular profiles among vegetarians and vegans in Central and Eastern Europe. Most studies in Poland have been online surveys with a limited number of participants following plant-based diets [33,34,35,36]. These studies frequently focus on potential deficiencies in critical nutrients such as protein, vitamin D, and vitamin B12. Recent findings from the national WOBASZ project revealed that flexitarian and vegetarian dietary patterns are not very common among Polish adults. The analysis indicated inappropriate intake of several nutrients, including potent bioactive compounds, regardless of the dietary regimen. Although the study involved a large cohort, the low percentage of individuals adhering to plant-based diets made it difficult to draw definitive conclusions [13]. Other studies have primarily assessed anthropometric variables, serum glucose, and lipid profiles, without a comprehensive evaluation of lifestyle factors and CVD risk factors, necessitating further research [35].

Therefore the aim of this study is to assess the nutritional quality and cardiovascular profiles of individuals following plant-based diets. Particular attention was paid to investigate adherence to current nutrition guidelines in vegetarians, vegans and omnivores as well as a relationship between dietary patterns and CVD outcomes. Unlike previous studies, this research will incorporate a comprehensive assessment of lifestyle characteristics and additional variables such as metabolic and inflammatory biomarkers.

## 2. Materials and Methods

### 2.1. Study Population

The study was designed and conducted at the Medical University of Lodz, Poland, within the Department of Preventive Medicine, the Students’ Association of Lifestyle Medicine and the Department of Sports Medicine. Written informed consent was obtained from all participants prior to their involvement in the study. The study protocol received approval from the Ethical Committee of the Medical University of Lodz (RNN/287/21KE). All clinical investigations were conducted in accordance with the principles outlined in the Declaration of Helsinki.

Participants were recruited through various channels, including posters, leaflets, and social media, targeting adult residents of the urban area of Lodz, with a specific emphasis on individuals following plant-based diets. These invitations were for a study examining the correlation between plant-based dietary patterns and selected health indicators. Participants were scheduled for examinations on specific days, with detailed information provided regarding all planned procedures, as well as their indications and contraindications. Upon completion of the study, participants received their examination results along with individualized recommendations from specialists within the research team.

### 2.2. Subjects

A total of 264 adult volunteers consented to participate in the project conducted between January 2022 and May 2023. For the purpose of this analysis, the subjects were categorized into three dietary groups: vegans (VG; *n* = 50; 33 women, mean age 33.3 years), vegetarians (VN; *n* = 101, 74 women, mean age 32.9 years) and omnivores (OV; *n* = 113, 44 women, mean age 53.2 years). Due to a substantial age difference between the VG and VN groups compared to the OV group, participants aged 50 years and older were excluded from the OV group, reducing its size from 113 to 66 subjects. Finally, the study population comprised 199 individuals (136 women and 63 men; mean age 33.9  ±  8.9 years) including 101 vegetarians (mean age 32.9 ± 11.5 years), 50 vegans (mean age 33.3 ± 8.5 years) and 48 omnivores (mean age 35.6 ± 6.8 years).

The classification of participants into the studied groups was based on dietary information obtained from nutritional interviews. Subjects were classified as vegetarians if they had excluded meat (including red meat, poultry, and fish) but consumed other animal products, such as eggs and dairy for at least 12 months. Those who reported excluding all animal products from their diet for at least 12 months were classified as vegans.

**Figure 1:**
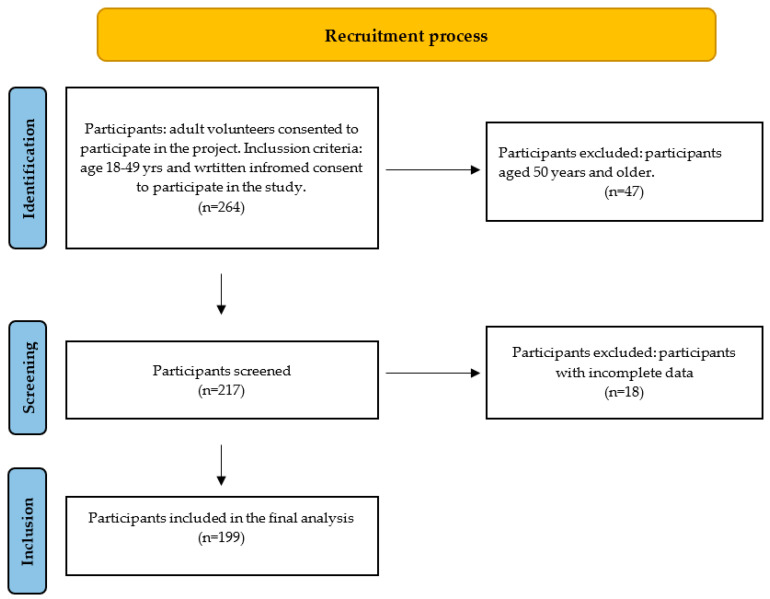
Flow chart of participant recruitment.

The invitation to the study was addressed to vegetarians and vegans, but the dietary interview showed that a part of the volunteers were meat-eaters. Therefore those who declared intake of meat, even occasionally, were categorized as omnivores. Figure 1 describes the flow of recruitment.

### 2.3. Protocol and Measures

Participants were instructed to report to the center between 7:30 and 9:00 a.m. after a minimum of 12 h of overnight fasting and rest. The study protocol involved a comprehensive assessment including a questionnaire interview, anthropometric and blood pressure measurements, endothelial function and a blood sample collection. The questionnaire covered various aspects such as sociodemographic characteristics, smoking status, dietary patterns, physical activity levels, and medical history. Additionally, the Healthy Lifestyle and Personal Control Questionnaire was performed [37].The procedures were conducted by the interdisciplinary team comprising laboratory diagnostician, nurses, dietitian, physiotherapists, students and physicians. All assessments and examinations were completed during a single visit.

### 2.4. Assessment of Dietary Patterns and Nutrition

Dietary patterns and the intake of macro- and micronutrients were evaluated using the Food Frequency Questionnaire (FFQ) and a 24-h dietary recall. The FFQ assessed usual frequency of consumption of selected food products over the past six months. The questionnaire included 88 food product groups, encompassing a wide range of items such as meat products, fish, dairy, eggs, different fats, vegetables, fruit, beverages as well fortified foods including calcium-fortified soy drinks and vitamin B12-fortified soy and oat drinks.. For each item, the participants indicated their consumption frequency using one of six options: no consumption, less than once a month, once to three times a month, one to three times a week, four to six times a week, or daily. The FFQ was based on the questionnaire used in our previous national studies [13]. Moreover, the usual supplementation was documented.

Daily food consumption was estimated through a 24-h dietary recall, in which respondents listed all the meals, beverages, additional food products consumed within 24 h prior to the study. Food items were categorized into groups according to their type and origin. The collected food records were analysed using Dieta 6.0 software (2018, Institute of Food and Nutrition, Independent Laboratory of Epidemiology and Nutrition Standards, Warsaw, Poland) and calorie chart databases. The intake of selected micronutrients were assessed according to the Polish Estimated Average Requirements by Jarosz et al. [38].

According to the analysis of the FFQ and 24-h dietary recall vegans did not declare intake of meat and animal-derived products. Vegetarians did not declare consumption of any meat (including poultry and fish), but they consumed other animal products (eggs, dairy, honey). Omnivores declared consumption of meat and other animal products with different frequency (from “less than once a month” to “everyday”).

### 2.5. Assessment of Cardiovascular Risk Factors

Sociodemographic data, medical history, nutritional habits, smoking status, physical activity level, knowledge of health behaviors, and quality of life were collected through structured interviews. The sociodemographic variables considered in this study included age, educational attainment, and marital status. Educational level was categorized as elementary, secondary, or university education.

Smoking status and physical activity were self-reported. Participants were classified as non-smokers if they had never smoked or were former smokers. Physical activity was assessed using the International Physical Activity Questionnaire (IPAQ short form).

Information on the history of hyperlipidemia, diabetes, hypertension, and medication use was obtained through the questionnaire.

Fasting blood samples were collected from the antecubital vein to measure several parameters. For this analysis, the following parameters were assessed: lipids profile, glucose, uric acid, and C-reactive protein (hsCRP). Enzymatic methods were used to determine serum total cholesterol, glucose, triglycerides, uric acid concentrations (COBAS INTEGRA 400 Plus, Roche^®^) (Roche Diagnostics, Warsaw, Poland). High-density lipoprotein cholesterol (HDL-C) was measured using the precipitation method. Low-density lipoprotein cholesterol (LDL-C) was calculated using the Friedewald formula. C-reactive protein was measured using the immunoturbidimetric method with tris buffer and anti-CRP antibodies. Blood pressure was measured three times on the left arm after 5-min of rest in the seated position using a manual sphygmomanometer(the SOHO 150 blood pressure monitor) and a stethoscope (PMT, Jankowice, Poland). The average from all three measurements was used for the analysis. Anthropometric measurements were obtained using standard methods. Body weight mass was measured to the nearest 100 g using calibrated scales (with participants wearing light indoor clothing and no shoes). Height was measured in the standing position using a stadiometer (without shoes), to the nearest 0.5 cm. Waist circumference was measured with an non-extensible metric tape at the midpoint between the lowest rib and the iliac crest (in underwear, standing position) to the nearest 0.5 cm. Body mass index (BMI) was calculated as body mass (kilograms) divided by square of height (meters), serving as an indicator of relative body weight and health status [39].

To achieve a comprehensive assessment of lifestyle and cardiovascular risk, the American Heart Association’s Life’s Simple 7 (LS7) tool was employed. The LS7 was developed in 2010 to define ideal cardiovascular health according to seven modifiable risk factors [40]. An overall LS7 score was calculated and categorized into “poor”, “intermediate”, and “ideal” cardiovascular health according to the guidelines. Specifically, as previously done [41] ideal levels for LS7 factors were defined as follows: non-smoker or having quit > 1 year ago; BMI < 25 kg/m^2^; ≥150 min/week of moderate or vigorous physical activity; 4 to 5 components of a healthy dietary pattern; untreated total cholesterol < 5.2 mmol/L; untreated blood pressure < 120/80 mm Hg; and untreated fasting glucose < 5.6 mmol/L. Poor LS7 factors included current smoking, BMI ≥ 30 kg/m^2^, no moderate or vigorous physical activity, 0 to 1 components of a healthy dietary pattern, total cholesterol ≥ 6.2 mmol/L, blood pressure ≥ 140/90 mm Hg, and fasting glucose ≥ 7.0 mmol/L, regardless of medication use. Intermediate conditions fell between ideal and poor. The LS7 score was calculated by assigning 2 points for ideal, 1 point for intermediate, and 0 points for poor status for each of the 7 factors, yielding a summary score ranging from 0 to 14, with higher scores indicating better health status.

### 2.6. Statistical Analysis

All the statistics were conducted using Statistica software version 13.1 (StatSoftPolska Sp. z o.o., 2024 update; www.statsoft.pl (accessed on 1 January 2024)). The distribution of variables was assessed with the Shapiro–Wilk test, and homogeneity of variances was evaluated using Levene’s test. Results are reported as means with standard deviations (SD) or medians with 95% confidence intervals (CIs). Categorical data are presented as counts and percentages. Differences between groups with normally distributed data were analyzed using the Student’s t-test. For non-normally distributed data, the Mann–Whitney U test was utilized. The chi-square test was employed to compare categorical variables. If the variables had a normal distribution, the differences among all three groups were assessed using one-way ANOVA on ranks, with the Dunn-Bonferroni post-hoc test for pairwise comparisons. Spearman’s rank correlation coefficient was used to explore relationships between selected variables. Statistical significance was set at *p* < 0.05.

## 3. Results

### 3.1. Baseline Data Analysis

Sociodemographic characteristics of the studied groups is presented in Table 1. Among vegetarians and vegans a substantial majority were single and possessed university degrees. In the OV group there were similar numbers of singles and married/in partnership. More than two thirds had university and 25% of the omnivores secondary attainment.

### 3.2. Analysis of Nutrition Quality According to the Dietary Patterns

Table 2 presents the daily intake of energy, macronutrients and fiber among the study participants. The average caloric intake was consistent across the VN, VG and OV groups. Carbohydrates constituted the predominant source of energy in all dietary patterns, followed by fats and proteins. Notably, vegans exhibited the lowest protein intake relative to the other groups (*p* < 0.05).

While total fat intake was comparable among the groups, variations were observed in the consumption of specific fatty acids. Monounsaturated fatty acids (MUFA) were the most frequently consumed across all dietary patterns (Table 2). Vegans consumed nearly twice as much MUFA relative to saturated fatty acids (SFA) and demonstrated a significantly higher intake of polyunsaturated fatty acids (PUFA) compared to the other groups. In contrast, SFA were the predominant fatty acids in the diets of omnivores and vegetarians, with vegetarians showing the highest daily intake of SFA. There were significant differences in dietary cholesterol intake, with vegetarians consuming the highest levels. Vegans had substantially lower cholesterol intake compared to both vegetarians (*p* < 0.05) and omnivores (*p* < 0.05). Additionally, vegans had approximately double the dietary fiber intake compared to omnivores (*p* < 0.05) and significantly more compared to vegetarians (*p* < 0.05) (Table 2).

According to the analysis of the FFQ and 24-h dietary recall vegans did not declare intake of meat and animal-derived products. Vegetarians did not declare consumption of any meat (including poultry and fish) while other animal products (eggs, dairy, honey) were eaten with different frequency (from “less than once a month” to “everyday”). Omnivores declared consumption of meat and other animal products.

Table 3 outlines the daily intake of micronutrients by dietary pattern. Omnivores declared lower intake of most of the analyzed nutrients as compared to VG and VN, with the most pronounced differences observed in phosphorus, magnesium, iron, manganese, vitamin A, C, E, vitamin B6, and folate. Conversely, OV had the highest intake of vitamin B12. Comparisons VN and VG revealed that vegans had significantly higher intakes of potassium, magnesium, manganese, vitamin C (*p* < 0.05) whereas vegetarians had higher intakes of phosphorus, calcium and vitamin B2 (Table 3).

Regarding regular supplements use there were important differences found among the studied groups. About 92% of vegans used supplements on daily basis, mainly vitamins B12, D3, zinc, magnesium and omega-3 fatty acids. Among vegetarians about 89.2% persons declared regular supplementation, mainly vitamin B12, D3, magnesium and omega-3 fatty acids. About 68.7% of OV took regularly supplements including vitamins D3 and C or multivitamin products.

Table 4 details adherence to nutritional recommendations. Deficiencies were noted in several nutrients across all dietary patterns, with significant shortfalls in potassium, niacin, and vitamin B12. Nevertheless, over 90% of vegetarians and vegans met the recommended intake levels for copper and manganese, and a substantial proportion adhered to the recommended levels for phosphorus, magnesium, vitamins A, E, and B6. Additionally, approximately 80% of vegans met the recommended intake for vitamin C. Overall, vegans demonstrated the highest level of adherence to nutritional guidelines, with significant differences noted between VN and OV. The majority of OV did not achieve the recommended intake levels for the analyzed micronutrients (Table 4).

### 3.3. Adherence to the AHA Life’s Simple Seven Criteria According to the Dietary Patterns

Overall, a substantial proportion of participants demonstrated favorable outcomes, particularly regarding serum cholesterol and glucose levels. Nonetheless, a significant percentage of both VN and OV fell into the poor category for smoking habits. Additionally, most participants reported inadequate levels of physical activity, with plant-based diet adherents showing particularly low activity levels.

Dietary analysis revealed that vegans achieved the highest scores, with 61.3% exhibiting ideal adherence to dietary recommendations. In contrast, over two-thirds of VN and OV demonstrated intermediate adherence to nutritional guidelines, with vegetarians showing the highest prevalence of poor dietary scores (Table 5).

The summary LS7 scores indicated that 86.9% of the study population achieved an ideal score, 13.1% attained an intermediate score, and none were classified as poor. Among the dietary patterns, vegans had the highest proportion of ideal scores (95.5%), followed by OV (85.2%) and VN (83.3%)

### 3.4. Cardiovascular Characteristics According to the Dietary Patterns

Table 6 summarizes the cardiovascular characteristics of the study groups. Mean values for anthropometric parameters did not differ significantly across the dietary patterns.

Analysis of the lipid profiles revealed that vegans had significantly lower levels of serum cholesterol, LDL cholesterol (LDL-C), and triglycerides (TG) compared to vegetarians (VN) and omnivores (OV). Specifically, the highest levels of total cholesterol and LDL-C were observed in the OV group relative to VN and VG (*p* < 0.001). Additionally, vegans exhibited significantly lower fasting glucose concentrations compared to OV (*p* < 0.05). Moreover, vegans had substantially lower hsCRP levels than VN and OV (*p* < 0.05).

No cases of overweight or obesity were observed among VN and VG participants, whereas OV included 9 cases of overweight and 2 cases of obesity. Additionally, no instances of impaired fasting glucose (IFG) or elevated blood pressure were noted among vegans.

Hypercholesterolemia was identified in 56.2% of OV participants, significantly more than the 26.7% in VN and 16.0% in VG (*p* < 0.05). Elevated blood pressure was recorded in one vegetarian and 6.2% of OV participants. The OV group also had a significantly higher incidence of IFG compared to VN and VG (25%, 3%, and 0%, respectively). Smoking prevalence was approximately 21% among VN and OV participants, and 6% among VG participants.

## 4. Discussion

Adequate nutrition plays a critical role in the prevention of chronic diseases, particularly CVD and metabolic disorders. Current dietary guidelines advocate for well-balanced, minimally processed plant-based diets as an effective strategy to enhance health outcomes, improve quality of life, and potentially reduce mortality rates [1,6,14,42,43]. Despite a global shift towards reducing or eliminating animal-derived products from daily nutrition, previous research from the WOBASZ study in Poland demonstrated a relatively low prevalence of plant-based dietary patterns (0.16% adherence to vegetarian diets and 8.8% flexitarianism among women, 5.7% among men) [13]. Notably, in that study, vegetarians and flexitarians exhibited dietary habits similar to omnivores, and no vegans were included, limiting conclusions about the cardiovascular impacts of stricter plant-based diets.

The current study aims to bridge this knowledge gap by providing a detailed analysis of the nutritional intake and cardiovascular health outcomes among omnivores, vegetarians, and vegans residing in a large urban area of Poland. This study is the first in the region to utilize a comprehensive, academically structured protocol with a relatively large cohort of vegans and vegetarians, enabling a more robust comparison of dietary patterns and their associations with cardiovascular profiles.

From a macronutrient perspective, all dietary groups demonstrated appropriate balance, though protein intake was expectedly lowest among vegans and highest among omnivores, consistent with previous research [8,28,29,30,44,45]. The proportion of energy derived from carbohydrates was highest in vegans, reflecting typical dietary characteristics of plant-based patterns, and corroborating findings from other international studies [8,13,28,29,30,31,44,45,46,47,48,49]. No significant differences in total fat consumption were observed between groups; however, saturated fat intake was unexpectedly highest among vegetarians, a finding that contrasts with many prior studies where plant-based diets were associated with lower intakes of SFA [13,28]. Cholesterol intake was also highest among vegetarians, whereas vegans exhibited markedly lower cholesterol consumption, aligning with the typical cholesterol-free nature of vegan diets [13,28,44,46].

Dietary fiber intake, a key factor in the prevention of CVD, was significantly higher among vegans, who consumed double the fiber compared to omnivores. Only 12% of omnivores met the daily fiber intake recommendation (≥30 g/day), compared to 30% of vegetarians and 48% of vegans, although overall fiber intake in all groups was lower than in other studies [28,30,44,45,46,47,49]. Fiber’s role in reducing blood pressure, cholesterol, and inflammatory markers, as well as its ability to decrease the risk of atherosclerosis, has been well-documented [2,50,51]. Thus, the markedly higher fiber intake observed among vegans may contribute to the more favorable cardiovascular profiles in this group.

In terms of micronutrient intake, omnivores demonstrated significantly lower intake of most vitamins and minerals compared to vegans, with the exception of calcium and vitamin D, which were highest in vegetarians. Vegans had higher intakes of micronutrients such as copper and manganese, while vegetarians showed greater consumption of calcium and vitamin B12, which is typically limited in plant-based diets unless fortified foods or supplements are used [13,28,45,46]. Adherence to dietary recommendations was generally suboptimal, particularly for vitamins A, C, E, folate, zinc, and iron in omnivores. Vegans and vegetarians also exhibited deficiencies, notably in vitamin D, B12, niacin, calcium, and potassium, although the results occurred more favorable than in previous Polish studies [13]. These findings highlight the importance of targeted nutritional education and supplementation for individuals following plant-based diets to prevent nutrient deficiencies that could compromise long-term cardiovascular health.

To assess cardiovascular health, the study employed the American Heart Association’s LS7 framework, a validated tool for evaluating adherence to ideal cardiovascular health metrics, including smoking status, physical activity, body mass index, diet, cholesterol, blood pressure, and fasting glucose levels. This study is the first to apply the LS7 criteria across different dietary groups in Poland, including vegans. Notably, 86.9% of participants achieved all seven ideal LS7 components, a far more favorable profile than reported in prior Polish and international studies, where the prevalence of ideal CVH ranged from 0.04% to 23% [52,53,54,55,56,57,58,59,60,61]. Vegans exhibited the highest percentage of ideal cardiovascular health (95.5%), followed by omnivores (85.2%) and vegetarians (83.3%). The most challenging LS7 metric to achieve was adherence to an ideal diet, particularly among vegetarians, while ideal cholesterol levels were achieved by 100% of participants, underscoring the significant cardiovascular benefits of plant-based diets.

The results of this study are consistent with a growing body of evidence supporting the cardioprotective effects of plant-based diets, particularly vegan diets, which have been associated with favorable lipid profiles, improved glycemic control, and lower risks of hypertension, obesity, and diabetes [25,62,63,64]. Vegan participants in this study demonstrated significantly lower levels of serum cholesterol, LDL-C, triglycerides, fasting glucose, and hs-CRP compared to omnivores and vegetarians. No cases of overweight, obesity, or hypertension were observed among vegans, whereas omnivores exhibited the least favorable cardiovascular profiles, with higher rates of hypercholesterolemia, smoking, and impaired fasting glucose. These findings align with prior meta-analyses demonstrating that plant-based diets are associated with lower BMI, serum cholesterol, and glucose concentrations, all of which are critical factors in reducing the incidence of ischemic heart disease and related cardiovascular events [1].

Emerging research also points to the anti-inflammatory effects of plant-based diets, largely due to their high content of fiber, polyphenols, and antioxidants from fruits, vegetables, and whole grains [65,66,67,68,69]. In this study, vegans exhibited significantly lower hs-CRP levels compared to other groups, reflecting reduced systemic inflammation, a key contributor to atherogenesis and cardiovascular events [70,71,72,73]. These findings corroborate previous intervention trials that have shown marked reductions in CRP and other inflammatory markers following the adoption of a vegan diet [72,74].

Despite the substantial evidence supporting the cardiovascular benefits of plant-based diets, adherence to optimal nutritional guidelines remains suboptimal in many populations, including Poland [13,75,76]. There is a need for improved public health strategies that focus on education and the promotion of plant-based diets, particularly in healthcare settings where dietary interventions could yield significant improvements in cardiovascular health outcomes [77,78,79,80]. Tailored programs, such as social prescribing or plant-based lifestyle interventions, have demonstrated promising results and could be further explored to enhance dietary adherence and long-term CVD prevention [79,81].

This study’s limitations include the use of self-reported dietary recalls and a non-random sample, which may affect generalizability. As the present study had a cross-sectional design, several confounding biases (selection, recall. etc.) might be possible. Moreover, most of the variables did not have a normal distribution which made deeper multivariate analyses difficult. Therefore, further studies on larger samples with randomized recruitment would be recommended with additional ANCOVA analysis.

However, the study strengths include a standardized methodology conducted by experienced professionals and a comprehensive cardiovascular assessment protocol, which offers valuable insights into the cardiovascular impacts of plant-based diets in an understudied Central and Eastern European population.

## 5. Conclusions

Our research indicates that plant-based diets are associated with a better cardiovascular profile compared to traditional diets, with vegan diets showing particularly favorable outcomes in terms of nutrition quality and lower serum LDL-C, triglycerides, and hs-CRP concentrations. High prevalence of smoking and hypercholesterolemia among both meat-eaters and vegetarians, as well as suboptimal intake of essential nutrients, underscores the need for more effective public health interventions and improved nutrition education.

## Figures and Tables

**Table 1:** Sociodemographic characteristics of the studied groups.

	Vegans *n* = 50	Vegetarians *n* = 101	Omnivores *n* = 48
Age, years (mean ± SD)	33.3 ± 8.5	32.9 ± 11.5	35.4 ± 685
Marital status, f (%)			
Single	35/50 (70.0)	69/101 (68.3)	21/48 (43.8)
Married/partnership	13/50 (26.0)	29/101 (28.7)	20/48 (41.6)
Divorced	1/50 (2.0)	3/101 (3.0)	5/48 (10.4)
Widowed	1/50 (2.0)	0/101 (0.0)	1/48 (2.1)
Educational level, f (%)			
Primary	1/50 (2.0)	0/101 (0.0)	4/48 (8.3)
Secondary	4/50 (8.0)	19/101 (18.8)	12/48 (25)
University	45/50 (90.0)	82/101 (81.2)	31/48 (64.6)

Data concerning marital status and educational level are presented as fractions (f) and percentages (%).

**Table 2:** Daily intake of energy, macronutrients, cholesterol and fiber in the studied groups.

	Vegans *n* = 50	Vegetarians *n* = 101	Omnivores *n* = 48
Energy, kcal	1693.9 (1678.9; 1958.4)	1679.4(1622.4; 1846.5)	1597.7 (1523.7; 1850.6)
Total protein, g	54.4 (54.7; 70.9)	67.7 ± 25.5 ^1^	77.1 ± 27 ^1,b^
Proteins (%)	13.8	15.6	18.3
Carbohydrates, g	256.1 (234.3; 284.5) ^a^	223.1(220.4; 255.5)	198.7 (187.2; 234.3) ^b,c^
Carbohydrates (%)	57.1	54.8	50.1
Total fat, g	63.7 ± 24.2 ^1^	57.7 (56.9; 66.9)	61.1 ± 24.8 ^1^
Fat (%)	31.6	32.1	32.5
SFA, g	8.5 (8.8; 14.4) ^a^	13.3 (14.1; 18.5)	9.2 (8.4; 14.2) ^b,c^
MUFA, g	20 (18.1; 25.8)	21.4 (20.4; 24.8)	8.5 (9.5; 17.2) ^b,c^
PUFA, g	14.7 (14.2; 19.7) ^a^	12 (11.9; 14.7)	4.9 (5.3; 9) ^b,c^
Cholesterol, mg	9.3 (3.2; 18.9) ^a^	86.6 (108.6; 168.8)	64.5 (67.8; 148.8) ^b,c^
Fibre, g	29.2 (27.8; 36.6) ^a^	23 (23.1; 27.1)	16 (13.7; 20.1) ^b,c^

^1^ means ± SD; in other cases data shown as medians (CI). Statistical significance between: Vegetarians vs. Vegans if ^a^ *p* < 0.05; Vegans vs. Omnivores if ^b^ *p* < 0.05; Vegetarians vs. Omnivores if ^c^ *p* < 0.05.Abbreviations: SFA—Saturated Fatty Acids; MUFA—Monounsaturated Fatty Acids; PUFA—Polyunsaturated Fatty Acids.

**Table 3:** Daily intake of micronutrients in the studied groups.

	Vegans	Vegetarians	Omnivores
Potassium, mg	3054.8 (2815.8; 3846.2)	2907.7 (2825.8; 3355.5)	1508.0 (1455.0; 2435.2) ^b,c^
Sodium, mg	1928.0 (1630.6; 2397) ^a^	2345.1 (2103.0; 2619.9)	872.2 (1163.9; 2034.9) ^b,c^
Phosphorus, mg	1068.3 (919.6; 1272)	1772.0 ± 500.4 ^1^	598.6 (526.4; 864.4) ^b,c^
Magnesium, mg	413.1 ± 208.6 ^1^	343.1 ± 140.8 ^1^	167.6 (157.9; 241.3) ^b,c^
Iron, mg	15.0	12.2 (10.8; 17.8)	7.8 (7.0; 10.7) ^b,c^
Calcium, mg	563.0 (521; 735.6) ^a^	723.6 (702.6; 894)	508.0 (464.7; 727.8) ^c^
Zinc, mg	8.9 ± 4.6 ^1^	8.7 ± 3.5 ^1^	4.4 (4.0; 6.3) ^b,c^
Copper, mg	2.1 (1.7; 2.4) ^a^	1.5 (1.5; 1.8)	0.8 (0.7; 1.1) ^b,c^
Manganese, mg	7.6 (6.5; 9.7) ^a^	4.8 (5.0; 6.5)	2.2 (2.6; 6.1) ^b,c^
Vitamin A, µg	704.5 (612.3; 971.5)	706.3 (687.8; 906.0)	223.0 (267.9; 549) ^b,c^
Vitamin C, mg	114.1 (108.5; 155.4)	107.5 (112.4; 148.6)	40.4 (51.0; 99.6) ^b,c^
Vitamin E, mg	10.4 (10.1; 15.1)	11.0 (10.4; 12.6)	2.5 (3.9; 8.1) ^b.c^
Vitamin D, µg	30.9 (30.3; 63.4)	32.6 (39.5; 60)	14.0 (18.4; 58.1) ^c^
Vitamin B1, mg	1.1 (1.0; 1.4) ^a^	0.9 (0.9; 1.4)	0.8 (0.7; 1.0) ^b,c^
Vitamin B2, mg	1.0 (0.9; 1.2) ^a^	1.3 (1.3; 1.5)	0.7 (0.7; 1.0) ^b,c^
Niacin, mg	11.9 (10.4; 15.7)	10.1 (9.8; 13.2)	6.7 (7.0; 12.3) ^b,c^
Vitamin B6, mg	1.6 (1.5; 2)	1.5 ± 0.6 ^1^	0.9 (0.8; 1.2) ^b,c^
Folate, µg	331.2 (256.7; 392.8)	324.0 (299.1; 364.9)	100.5 (132.6; 247.8) ^b,c^
Vitamin B12, µg	0.2 (1.2; 1.8) ^a^	1.5 (1.6; 2.2)	0.7 (1.1; 1.7) ^b^

^1^ means ± SD; in other cases data shown as medians (CI). Statistical significance between: Vegetarians vs. Vegans if ^a^ *p* < 0.05; Vegans vs. Omnivores if ^b^ *p* < 0.05; Vegetarians vs. Omnivores if ^c^ *p* < 0.05.

**Table 4:** Percentage of persons with the adequate intake of selected micronutrients according to the requirements.

	Recommended Daily Intake (Males/Females)	Vegans	Vegetarians	Omnivores
			N (%)	
Potassium, mg	≥4700	9 (18.0)	12 (11.9)	2 (4.2) ^c^
Calcium, mg	19–50 years ≥ 800; above 50 years ≥ 1000	15 (30.0)	42 (41.6)	14 (29.2)
Phosphorus, mg	≥700	36 (72.0)	81 (80.2) ^b^	18 (37.5) ^c^
Magnesium, mg	≥350/265	34 (68.0)	66 (65.4) ^b^	12 (25.0) ^c^
Iron, mg	≥18/10	28 (56.0)	52 (51.5) ^b^	11 (22.9) ^c^
Zinc, mg	≥11/8	24 (48.0)	36 (35.6) ^b^	9 (18.8) ^c^
Copper, mg	≥0.7	48 (96.0)	91 (90.0) ^b^	25 (52.1) ^c^
Manganese, mg	2.3/1.8	45 (90.0)	92 (91.1) ^b^	25 (52.1) ^c^
Vitamin A, µg	≥630/500	30 (60.0)	65 (64.4) ^b^	13 (27.1) ^c^
Vitamin E, mg	≥10/8	32 (64.0)	74 (73.3) ^b^	13 (27.1) ^c^
Vitamin B1, mg	≥1.1/0.9	30 (60.0) ^a^	45 (34.7)	15 (31.3) ^c^
Vitamin B2, mg	≥1.1/0.9	28 (56.0) ^a^	70 (69.3) ^b^	19 (39.6)
Niacin, mg	≥16/14	12 (24.0)	19 (18.8)	9 (18.8)
Vitamin B6, mg	≥1.1/1.3	35 (70.0)	63 (62.4) ^b^	19 (39.6) ^c^
Vitamin C, mg	≥75/60	40 (80.0)	79 (79.8) ^b^	21 (43.8) ^c^
Folate, µg	≥320	26 (52.0)	52 (51.5) ^b^	10 (20.8) ^c^
Vitamin B12, µg	≥2	8 (16.0) ^a^	37 (36.6)	11 (22.9)
Vitamin D, µg	≥15 ug (600 IU)	23 (46) ^a^	69 (68.3) ^b^	22 (45.8)

Statistical significance between: Vegetarians vs. Vegans if ^a^ *p* < 0.05; Vegans vs. Omnivores if ^b^ *p* < 0.05; Vegetarians vs. Omnivores if ^c^ *p* < 0.05.

**Table 5:** Adherence to the American Heart Association Life’s Simple 7 criteria in the studied groups.

LS7	Diet	PA	Smoking	BMI	TC	Glucose	BP
Vegan, f (%)							
Poor	0/49 (0.0)	6/50 (12.0)	3/50 (6.0)	2/50 (4.0)	0/50 (0.0)	0/50 (0.0)	1/45 (2.2)
Intermediate	19/49 (38.7)	27/50 (54.0)	0/50 (0.0)	6/50 (12.0)	0/50 (0.0)	0/50 (0.0)	15/45 (30.3)
Ideal	30/49 (61.3)	17/50 (34.0)	47/50 (94.0)	42/50 (84.0)	50/50 (100.0)	50/50 (100.0)	29/45 (64.5)
Vegetarians, f (%)							
Poor	26/99 (26.3)	11/101 (10.9)	21/101 (20.8)	1/101 (1.0)	0/101 (0.0)	1/101 (1.0)	4/98 (4.1)
Intermediate	62/99 (62.6)	43/101 (42.6)	0/101 (0.0)	18/101 (17.8)	0/101 (0.0)	3/101 (3.0)	34/98 (34.7)
Ideal	11/99 (11.1)	47/101 (46.5)	80/101 (79.2)	82/101 (81.2)	101 (100.0)	97/101 (96.0)	60/98 (61.2)
Omnivores, f (%)							
Poor	3/47 (6.4)	6/43 (14.0)	10/47 (21.3)	3/44 (6.8)	0/46 (0.0)	0/46 (0.0)	1/31 (3.2)
Intermediate	33/47 (70.2)	13/43 (30.2)	0/47 (0.0)	8/44 (18.2)	0/46 (0.0)	3/46 (6.5)	8/31 (25.8)
Ideal	11/47 (23.4)	24/43 (55.8)	37/47 (78.7)	33/44 (75.0)	46/46 (100.0)	43/46 (93.5)	22/31 (71.0)

Data presented as fractions (f) and percentages (%); Abbreviations: BP—Blood pressure, BMI—body mass index, LS7—Life’s Simple 7 score, PA—physical activity, TC—total cholesterol.

**Table 6:** Cardiovascular characteristics in the studied groups.

	Vegans	Vegetarians	Omnivores
BMI (kg/m^2^)	22.2 ± 2.4 ^1^	22.5 (21.6; 24.9)	23.0 (22.2; 24.5)
Waist circumference (cm)	75.0 (74.1; 82)	75.0 (74.7; 78.5)	77.0 (77.7; 85.2)
SBP (mmHg)	110.0 (105.6; 112.7)	110.0 (104.6; 111.2)	100.0 (102.5; 112.4)
DBP (mmHg)	70.0 (64.3; 68.9)	65.0 (63.1; 68.1)	60.0 (62.0; 67.9)
TC (mmol/L)	4.1 (4.1; 4.5) ^a^	4.5 (4.4; 4.8)	5.2 ± 1.0 ^1,b,c^
LDL-C (mmol/L)	2.3 (2.3; 2.7) ^a^	2.6 (2.6; 2.8)	3.1 ±0.8 ^1,b,c^
HDL-C (mmol/L)	1.5 ± 0.4	1.5 ± 0.5 ^1^	1.6 (1.5; 1.8) ^b^
TG (mmol/L)	0.7 (0.7; 0.9) ^a^	0.9 (0.9; 1.1)	0.9 (0.9; 1.1) ^b^
Glucose (mmol/L)	4.7 ± 0.3 ^1^	4.8 (4.7; 4.9)	4.8 (4.8; 5.0) ^b^
HbA1c (%)	5.0 ± 0.2 ^1^	5.00 (5.0; 5.1)	5.1 (4.2; 7.4)
hsCRP(mg/L)	0.3 (0.4; 1.1) ^a^	0.56 (0.6; 1.9)	0.6 (0.8; 4.2) ^b^

^1^ means ± SD; in other cases data shown as medians (CI). Statistical significance between: Vegetarians vs. Vegans if ^a^ *p* < 0.05; Vegans vs. Omnivores if ^b^ *p* < 0.05; Vegetarians vs. Omnivores if ^c^ *p* < 0.05. Abbreviations: BMI—Body Mass Index; LDL-C—low density lipoprotein cholesterol; HDL-C—high density lipoprotein cholesterol; TG—triglycerides; HbA1c—glycated haemoglobin; hsCRP—high-sensitivity C-reactive protein. Abbreviations: DBP—Diastolic blood pressure, SBP—Systolic blood pressure, TC—total cholesterol.

## Data Availability

Data supporting reported results may be available from the corresponding author upon reasonable request.
